# Grafting on a Non-Transgenic Tolerant Tomato Variety Confers Resistance to the Infection of a *Sw5*-Breaking Strain of *Tomato spotted wilt virus* via RNA Silencing

**DOI:** 10.1371/journal.pone.0141319

**Published:** 2015-10-23

**Authors:** Roberta Spanò, Tiziana Mascia, Richard Kormelink, Donato Gallitelli

**Affiliations:** 1 Dipartimento di Scienze del Suolo della Pianta e Degli Alimenti, Università degli Studi di Bari Aldo Moro, Via Amendola 165/A, Bari, Italy; 2 Istituto di Virologia vegetale del CNR, Unità Operativa di Supporto di Bari, Via Amendola 165/A, Bari, Italy; 3 Laboratory of Virology, Wageningen University, Droevendaalsesteeg 1, Wageningen, The Netherlands; University of Queensland, AUSTRALIA

## Abstract

RNA silencing controls endogenous gene expression and drives defensive reactions against invasive nucleic acids like viruses. In plants, it has been demonstrated that RNA silencing can be transmitted through grafting between scions and silenced rootstocks to attenuate virus and viroid accumulation in the scions. This has been obtained mostly using transgenic plants, which may be a drawback in current agriculture. In the present study, we examined the dynamics of infection of a resistance-breaking strain of *Tomato spotted wilt virus* (RB-TSWV) through the graft between an old Apulian (southern Italy) tomato variety, denoted Sl-Ma, used as a rootstock and commercial tomato varieties used as scions. In tests with non-grafted plants, Sl-Ma showed resistance to the RB-TSWV infection as viral RNA accumulated at low levels and plants recovered from disease symptoms by 21 days post inoculation. The resistance trait was transmitted to the otherwise highly susceptible tomato genotypes grafted onto Sl-Ma. The results from the analysis of small RNAs hallmark genes involved in RNA silencing and virus-induced gene silencing suggest that RNA silencing is involved in the resistance showed by Sl-Ma against RB-TSWV and in scions grafted on this rootstock. The results from self-grafted susceptible tomato varieties suggest also that RNA silencing is enhanced by the graft itself. We can foresee interesting practical implications of the approach described in this paper.

## Introduction

In plants, RNA silencing (RNA interference, RNAi) drives multiple regulatory and defensive reactions triggered by either endogenous or invasive double-stranded RNAs (dsRNA), which are diced into 21 to 24 –long ribonucleotide fragments by Dicer-like (DCL) endoribonucleases [1, 2]. From these fragments, generally called primary small RNAs (sRNAs), one strand becomes incorporated into members of the Argonaute (AGO) protein family to form an RNA-induced silencing complex (RISC) that starts to survey and cleave endogenous or invasive nucleic acids with sequence complementarity to the uploaded RNA strand. In turn, plant RNA-dependent RNA polymerases (RDRs) convert aberrant or functional RNA transcripts in a sRNA (in)dependent manner into dsRNAs, thereby increasing the amount of DCL substrate and leading to the production of secondary sRNAs and amplification of the RNAi signal. The RNAi pathway is harnessed with different DCLs, AGOs and RDRs that enables to control the expression of endogenous genes, transposons, repetitive DNA sequences, transgenes, viruses and viroids [1–7].

It is now well established that in virus-infected plants virus-specific sRNAs, like the virus, also follow the phloem transport and that in grafted plants they can be transmitted from a silenced rootstock to a non-silenced scion and vice-versa to trigger antiviral defense in recipient cells [8, 9]. With self-grafting experiments the grafting itself may enhance RNAi, as demonstrated by Han & Grierson [10] who showed that a weak-silencing state of the scion grafted onto a strong silencer stock could be reverted into a strong-silencing condition by the quick and massive release of sRNAs accumulated at the grafting junction, once the phloem between the scion and stock re-connected. Virus-derived sRNAs produced in the rootstock and transported in a susceptible scion through the graft junction may thus counteract the accumulation of viral RNA and reduce disease symptom expression in the recipient scion. This is also supported by a study from Kasai and colleagues [11] in which sRNAs produced in a transgenic rootstock expressing a non-infectious hairpin derived from the *Potato spindle tuber viroid* (PSTVd) moved through the graft junction into a non-transgenic scion where they counteracted a *de novo* PSTVd infection. Similarly, Ali *et al*. [12] reported that sRNA produced in a transgenic tobacco rootstock with silenced *NtTOM1* and *NtTOM3* genes, required for tobamovirus replication, moved into grafted scions of non transgenic tobacco plants that in turn became resistant to tobamovirus infection through the sRNA-induced silencing of *NtTOM1* and *NtTOM3*. Grafting thus may offer an additional or alternative, ready-to-use and flexible solution to control plant diseases caused by viruses in those cases where resistance (gene) strategies are limited or start failing.

*Tomato spotted wilt virus* (TSWV) is the representative of the *Tospovirus* genus within the arthropod-born *Bunyaviridae* [13] and one of the most detrimental pathogens of tomato and a wide range of vegetable crops. In tomato, TSWV disease symptoms range from mild to leaf necrosis and up to plant death depending on tomato genotype, viral isolate, developmental stage of the plant and environmental conditions [14]. TSWV is transmitted in a persistent propagative manner by several species of thrips (Family *Thripidae*, Genera *Frankliniella* and *Thrips*) [13], which, together with the enormous wide range of host plants, make it difficult to control [15, 16]. The viral genome consists of three negative-sense/ambisense RNA segments that according to the size are denoted large (L), medium (M) and small (S). While the L RNA is of entire negative polarity and codes for a putative RNA-dependent RNA polymerase (RdRp, L protein) in viral complementary (vc) sense, both M and S RNA are ambisense and code for the non-structural (NSm) cell-to-cell movement protein and the precursor (GP) to the surface glycoproteins Gn and Gc, respectively the nucleocapsid protein N and a second non-structural (NSs) protein with RNA silencing suppressor (RSS) activity [17]. The tospoviral NSs protein exhibits affinity to long dsRNA, small interfering (si)RNAs and micro-RNA (miRNA)/miRNA* duplexes [18]. *In vitro* TSWV NSs protein inhibits cleavage of long dsRNA by Dicer enzymes while *in vivo* the protein is able to interfere in the miRNA pathway as shown by suppression of miRNA-induced silencing of a GFP (eGFP) miRNA sensor construct [18]. These data supported the idea that tospoviruses interfere in the RNA silencing pathway by sequestering long dsRNA and/or small RNAs to prevent their cleavage by dicer/DCL and subsequent loading into (si/mi)RISC complexes. Accumulation of NSs protein in plants has also been observed to coincide with increased virulence of the virus [19].

The most successful strategy to control tospoviruses is the cultivation of resistant cultivars. Currently two single dominant resistance (*R*) genes are applicable for commercial resistance breeding against tospoviruses, i.e. *Tsw* and *Sw-5*. The first one originates from distinct *Capsicum chinense* accessions [20] and is highly specific as it only confers resistance against TSWV isolates [21]. Recently, *Tsw* has been shown to be triggered by the TSWV RNA silencing suppressor protein NSs [22]. The second one, *Sw-5* [23–25], is the most interesting one as it confers a broad tospovirus resistance against TSWV and various other tospoviruses. The resistance derives from the Stevens tomato cultivar obtained in South Africa by a cross between *Solanum peruvianum* and *S*. *lycopersicum* [26] and has recently been demonstrated to be triggered by the viral cell-to-cell movement protein NSm [27, 28]. In addition to breeding for resistance, genetically engineered resistance strategies have been deployed, which mostly involved the transformation of (partial) N gene sequences to confer RNAi-mediated resistance against tospoviruses with sequence complementarity to the transgene [29]. Despite of the efforts to obtain genetically resistant tomato cultivars, only the *Sw-5* dominant gene has been transferred in commercial tomato cultivars, making them resistant to common TSWV strains but not to the *Sw-5* resistance-breaking (RB) isolates that emerged recently in several tomato cropping areas ([30] and references therein).

In the framework of a regional project on biodiversity, we rescued seeds of the Manduria tomato cultivar (*Solanum lycopersicum* cv Manduria, Sl*-*Ma) grown in the past for its high tolerance to drought and used mainly for winter consumption in the Apulia region (southern Italy) due to its incredibly long shelf-life. In preliminary screening tests, plants of this tomato variety showed resistance to the infection of an RB isolate of TSWV and recovered from disease symptoms but the underlying mechanism of this resistance, is not yet known. In this study we show that RNAi is involved in the resistance to a *Sw-5* RB strain of TSWV and that such resistance can be induced in otherwise highly susceptible tomato scions when grafted onto a non-transgenic tomato rootstock from Sl*-*Ma. Resistance to TSWV infection seems to be the result of a combination of the ability of the rootstock to activate and hold a strong defense response based on RNAi and the graft itself as some levels of resistance were observed also in self-grafted susceptible genotypes. Since other documented cases of RNAi-mediated resistance to virus infection through grafting involved transgenic rootstocks, this non-transgenic approach presents an attractive practical application to limit the occurrence of RB strains of TSWV in tomato crops. Interestingly resistance to RB strains of TSWV was observed also in (the otherwise susceptible) genotypes of commercial tomato varieties carrying the *Sw-5* gene, when grafted onto Sl*-*Ma.

## Results

### Grafting and self-grafting enhance resistance to virus infection in tomato plants

**Fig 1:**
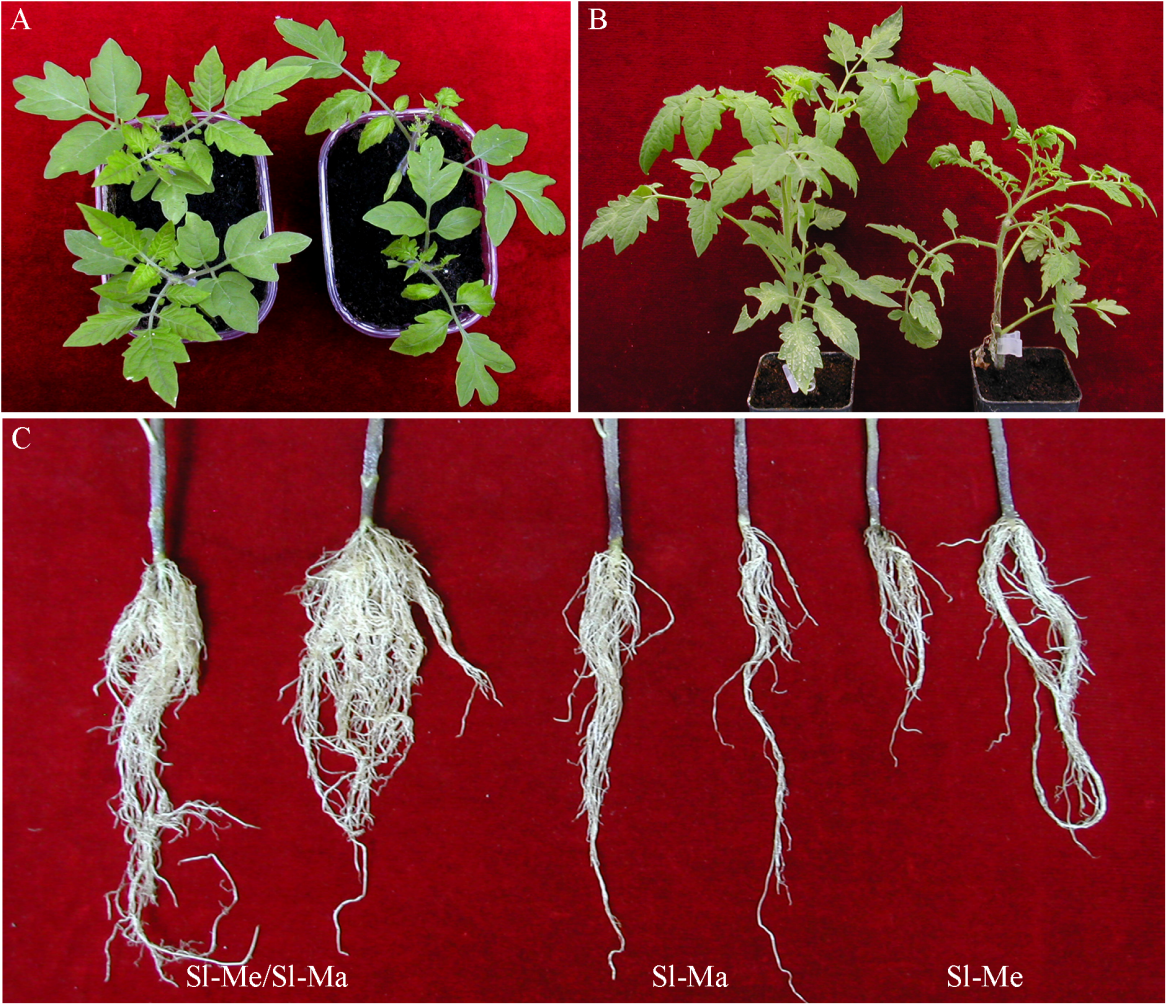
Differential response of grafted and non-grafted tomat\o genotypes to the infection of TSWV-CiPz. (**A**) Recovery from disease symptoms shown by Sl*-*Ma (left) but not from Sl*-*UC (right) at 28 dpi with TSWV-CiPz. (**B**) disease symptoms shown by Sl-Me (left) and Sl-Pu (right) grafted on Sl*-*Ma at 21 dpi with TSWV-CiPz. Sl-Me is a tomato variety carrying the *Sw-5* resistance gene to standard strains of TSWV. Sl-Pu is a tomato variety susceptible also to ordinary strains of TSWV. (**C**) Root development in plants of Sl-Me grafted on Sl*-*Ma compared with roots of non-grafted plants of Sl*-*Ma and Sl-Me.

**Fig 2:**
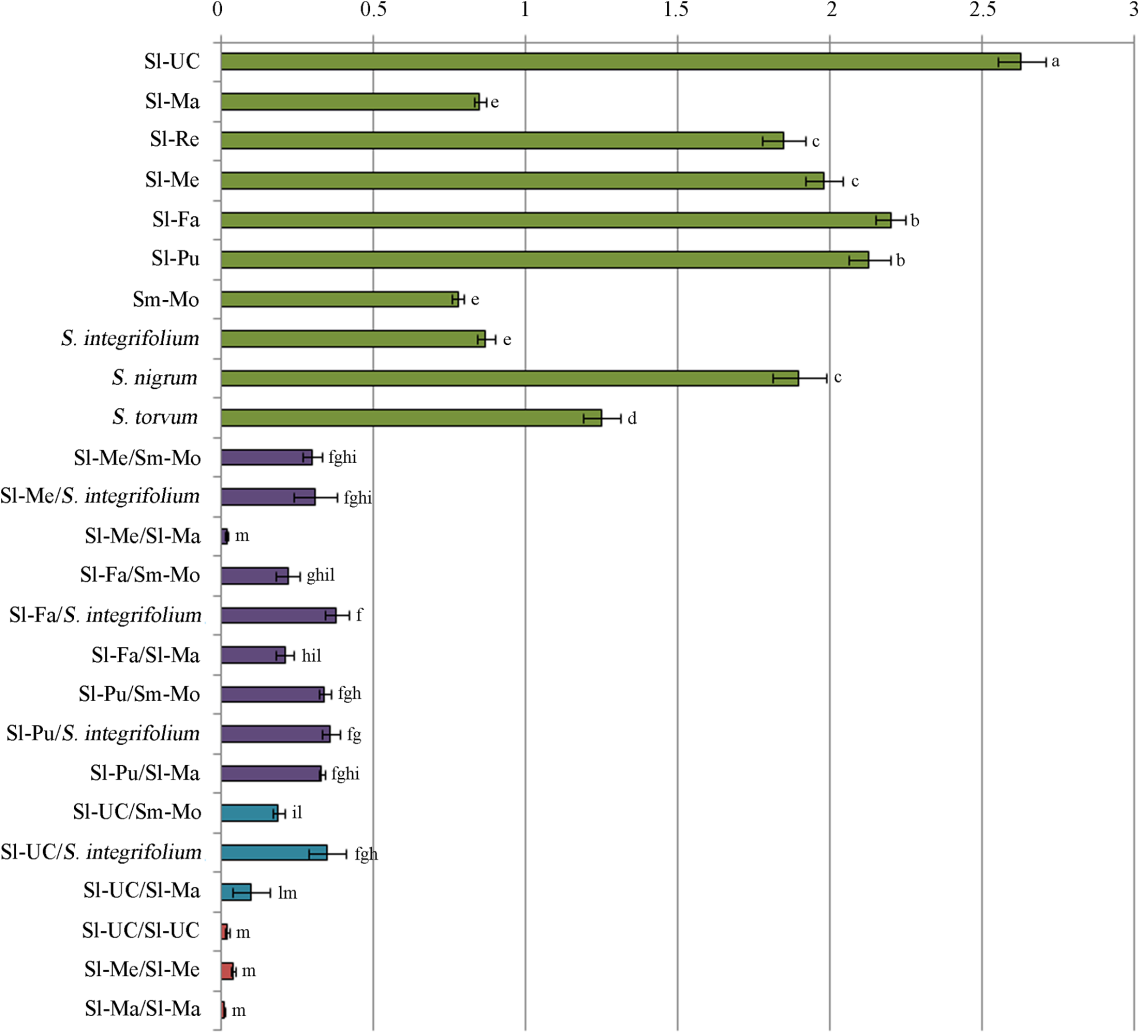
Accumulation of TSWV-CiPz RNA varies among Solanum genotypes and grafted and self-grafted plants. Load of viral RNA was estimated in *Solanum* spp genotypes (green bars), grafted (scion/rootstock) plants (violet bars) and self-grafted plants (blue bars) at 21dpi, by quantitative dot blot hybridization. Bars represent means of two independent experiments of spot intensity values of RNA M and were calculated on the basis of a standard curve generated by serial dilutions of a plasmid preparation containing the fragment of the RNA M targeted by the probe. Each bar represents average of three biological replicates for each of the two experiments and error bars on lines represent the standard error among replicates. Acronyms and symbols as in Table 1.

Prior to analyzing the effects of various tomato cultivar rootstocks on virus accumulation in scions, different So*lanum* spp genotypes where challenged with TSWV-CiPz and the accumulation of viral RNA analyzed. The following wild *Solanum* spp and local ecotypes of tomato and eggplant were selected: *Solanum melongena* cv Molfettese (Sm-Mo), *S*. *integrifolium*, *S*. *nigrum* (black nightshade), *S*. *torvum* (Turkey berry), *S*. *lycopersicum* cv Manduria (Sl-Ma) and *S*. *lycopersicum* cv Regina (Sl-Re). Messapico (Sl-Me) and Faino (Sl-Fa) were selected as commercial tomato varieties carrying the *Sw-5* gene, while Pullrex (Sl-Pu) and UC82 (Sl-UC) were used as susceptible controls. Upon infection with TSWV-CiPz, an *Sw-5* resistance breaking (RB) strain, *Solanum nigrum* and Sl-UC showed symptoms ranging from severe mosaic to leaf and stem necrosis and plant death, while those of Sl-Ma, Sm-Mo, *S*. *torvum* and *S*. *integrifolium* mostly consisted of mild mosaic (Table 1). Between 21 and 28 dpi Sl-Ma, Sm-Mo and *S*. *integrifolium* fully recovered from viral disease symptoms (Table 1, Fig 1A), while no recovery was observed with the other genotypes. Accumulation of viral RNA estimated in systemically infected leaf samples from three plants collected for each *Solanum* spp genotype showed differences congruent with the symptom severity observed (Table 1 and Fig 2). The lowest amount of viral RNA was detected in *S*. *integrifolium*, Sl-Ma and Sm-Mo (Fig 2) that were selected to be used in graft combinations with the four commercial tomato varieties Sl-UC, Sl-Me, Sl-Fa and Sl-Pu. Self-grafted plants of Sl-Me, Sl*-*UC and Sl-Ma served as control. The mildest symptoms were observed when Sl-Me and Sl-Fa scions, both carrying the *Sw-5* gene, were grafted on the Sl-Ma genotype. Plants recovered from disease symptoms by 21 dpi (Table 1 and Fig 1B and 1C) and by 28 dpi displayed approx 4-fold increased leaf canopy (1.996 ± 0.330 g from six grafted infected plants *versus* 0.442 ± 0.192 g from six non grafted plants) and 1.8-fold increased root development (1.203 ± 0.575 g from six grafted infected plants *versus* 0.660 ± 0.34 g from six non grafted plants). Analysis of the viral RNA load in samples of all graft combinations collected at 21 dpi revealed a substantial correlation with the severity of disease symptoms displayed by the scion. Compared to non grafted infected plants, approx 99% reduction in viral RNA accumulation was detected in the Sl-Me scion grafted on Sl*-*Ma rootstock as well as in all self-grafted plants (Fig 2).

**Table 1:** Disease symptoms observed in the *Solanum* spp genotypes and in grafted plants upon infection with TSWV-CiPz.

	Systemic Symptoms^a^			
**Solanum genotypes**	**7 dpi** ^b^	**14 dpi**	**21 dpi**	**28 dpi**
Sl-UC	Npp	VN, LE	LY	N, PD
Sl-Ma	MMos	MLD	R	R
Sl-Re	Mos	VN, LE	LY	N, PD
Sl-Pu	Npp	VN, LE	SLD, LY, LE	N, PD
Sl-Me	Npp	VN, LE	SLD, LY, LE	N, PD
Sl-Fa	Npp	VN, LE	SLD, LY, LE	N, PD
Sm-Mo	MMos	MMos	R	R
*S*. *integrifolium*	MMos	MMos	R	R
*S*. *nigrum*	Mos	SMos, SLD	N, SLD, LE	N
*S*. *torvum*	MMos	MMos	MMos	MMos
**Grafted plants** ^c^				
Sl-Me / Sm-Mo	MMos	SMos,	SMos, LY	LY, SLD
Sl-Me / *S*. *integrifolium*	Mos	SMos,	SMos, LY	LY, SLD
Sl-Me / Sl-Ma	MMos	MMos	R	R
Sl-Fa / Sm-Mo	MMos	Mos	Mos	R
Sl-Fa / *S*. *integrifolium*	Npp	LE, VN	LY, VN	N
Sl-Fa / Sl-Ma	MMos	Mos	R	R
Sl-Pu / Sm-Mo	Mos	SMos	SLD, VN	N
Sl-Pu / *S*. *integrifolium*	Npp	LE, VN	LY, VN	SLD, VN
Sl-Pu / Sl-Ma	Mos	SMos	LE, VN	LY, VN
Sl-UC / Sm-Mo	MMos	Mos	R	R
Sl-UC / *S*. *integrifolium*	SMos	SMos, LE	SLD, LY	N
Sl-UC / Sl-Ma	MMos	Mos	R	R
Sl-Me / Sl-Me	MMos	MMos	R	R
Sl-UC / Sl-UC	MMos	MMos	R	R
Sl-Ma / Sl-Ma	VMMos	VMMos	R	R

Sl-UC = *Solanum lycopersicum* Sl-Ma = *S*. *lycopersicum* 'Manduria'; Sl-Re = *S*. *lycopersicum* 'Regina'; Sl-Me = *S*. *lycopersicum* ' Messapico'; Sl-Fa = *S*. *lycopersicum* 'Faino'; Sl-Pu = *S*. *lycopersicum* Pullrex; Sm-Mo = *S*. *melongena* 'Molfettese'.

^a^Systemic symptoms are denoted with LE = leaf epinasty; LY = whole leaf blade yellowing; MLD = mild leaf distrotion; MMos = mild mosaic; Mos = mosaic; N = whole leaf necrosis; Npp = necrotic pin points; PD = plant death; R = Recovery; SLD = severe leaf distortion; SMos = severe mosaic; VN = vein necrosis.

^b^Days post inoculation (dpi).

^c^Grafted plants are indicated by name of the plant used as scion / name of the plant used as rootstock.

**Fig 3:**
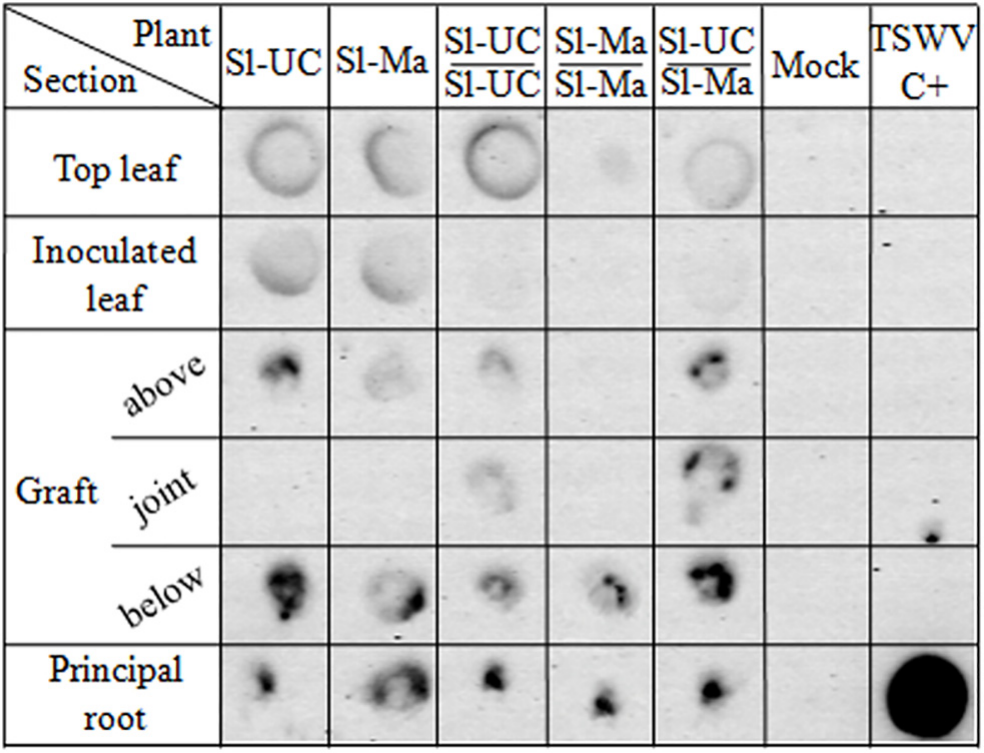
Localization of TSWV-CiPz RNA in grafted tomato plants. Localization of TSWV-CiPz RNA at 19 dpi by tissue print hybridization in cross sections of principal root, below, point and above the graft junction, inoculated leaf and top leaf of Sl*-*UC (UC82), Sl*-*Ma (Mand) and graft combinations. Mock = negative control plants mock-inoculated with buffer. TSWV C+ = plasmid preparation (50 ng) containing the fragment of the RNA M targeted by the probe.

Virus RNA distribution and accumulation in grafted and non-grafted plants was further analyzed by tissue print hybridization in cross sections of roots, stems, petioles and leaves (Fig 3). At 19 dpi, TSWV-CiPz RNA was detected in all sections but the hybridization signal was much weaker in the upper parts of the plants than in the roots and this was particularly evident in self-grafted Sl*-*Ma plants, where the hybridization signal in the top leaf was very weak.

### Presence of sRNAs specific to TSWV-CiPz in the Sl-Ma rootstock

**Fig 4:**
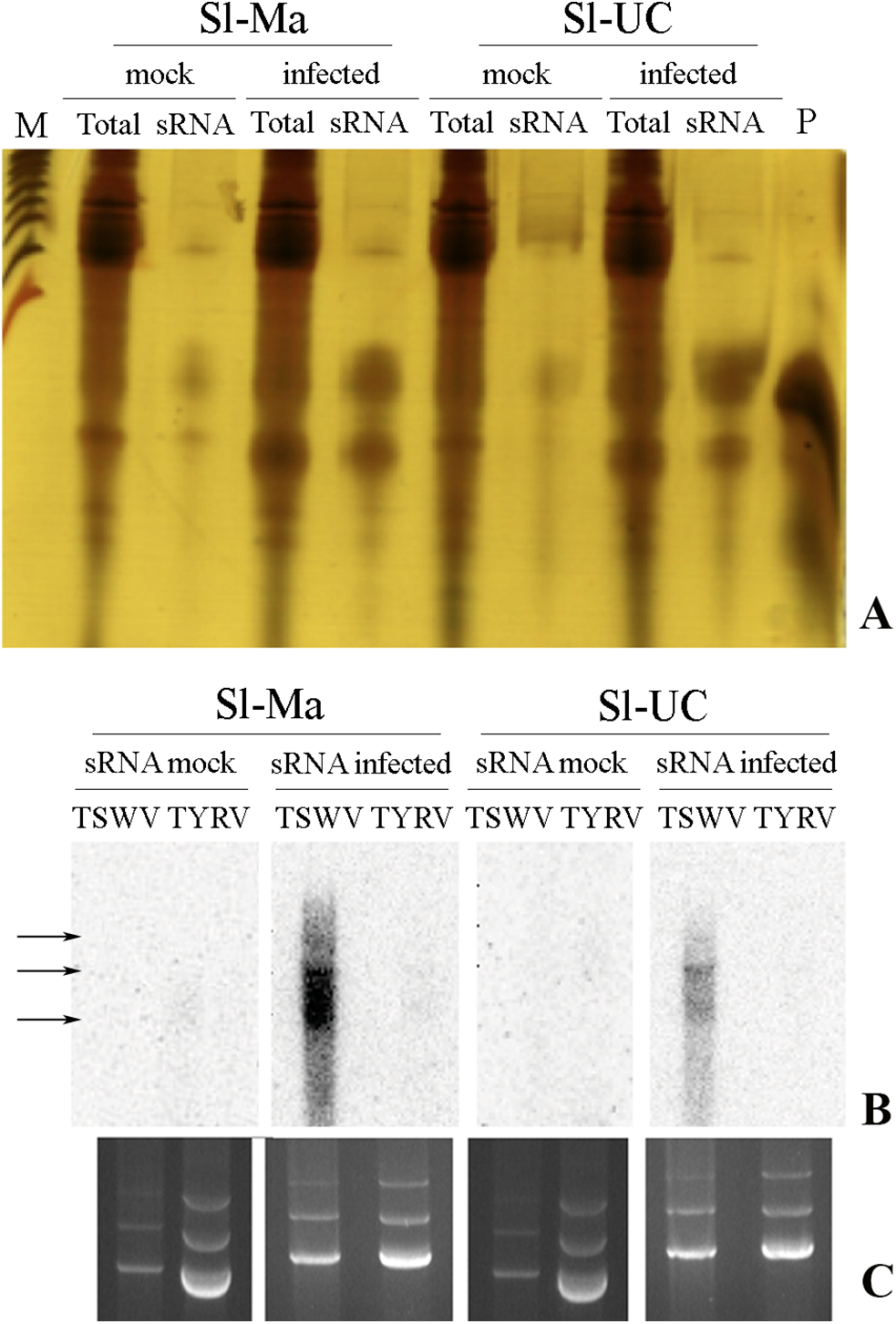
Detection of sRNA and mapping on the TSWV-CiPz genome. (**A**) silver staining of the total RNA (Total) and the sRNA-enriched preparations (sRNA) extracted from Sl-Ma and Sl-UC plants mock-inoculated (mock) or at 21 dpi with TSWV-CiPz (infected). M is a 100 bp DNA ladder (Promega, Madison, WI, USA). P is a custom 21 bp RNA oligonucleotide used as marker. (**B**) Northern blots of RNA extracted from purified preparations of TSWV-CiPz and TYRV hybridized with siRNA probes derived from the sRNA-enriched preparations obtained from Sl-Ma and Sl-UC plants mock-inoculated (mock) or at 21 dpi with TSWV-CiPz (infected) (see panel A). (**C**) ethidium bromide staining of RNA extracted from purified preparations of TSWV-CiPz and TYRV prior to transfer onto nylon membranes, demonstrating equal loading. Hybridization signals of sRNAs labeled with (γ-^32^P) ATP with virus genomic RNAs S, M and L pointed by arrows are visible only in RNA preparations from TSWV.

The next step was to evaluate whether an RNAi-based response could be involved in the different susceptibility to TSWV-CiPz observed among the *Solanum* genotypes tested and whether Sl-Ma has a better propensity than Sl-UC to mount such an RNAi-based response. When Sl*-*Ma and Sl*-*UC were challenged with the virus and low-molecular-weight RNAs purified at 21 dpi, a detectable increase in the population of sRNAs was clearly seen in both genotypes upon viral infection compared to mock-inoculated control plants (Fig 4). For a better characterization, sRNAs were isolated from a fixed amount of Sl*-*Ma and Sl*-*UC*-*infected plants and, after gel purification, end-labeled with [γ-^32^P] dATP and used in Northern blot hybridization on a standardized amount of viral RNA extracted from a preparation of TSWV-CiPz ribonucleoproteins (RNPs) produced and purified according to the protocol of Feldhoff *et al*. [31]. In analogy, and as a control, RNA was extracted from viral RNPs of the distantly related tospovirus *Tomato yellow ring virus* (TYRV), grown and purified according the protocol of Hassani-Mehraban *et al*. [32]. The radiolabeled sRNAs hybridized with all three viral RNAs of TSWV-CiPz but the hybridization signal with the sRNA probe derived from infected Sl*-*Ma plants was stronger than the signals obtained with the sRNA probe from Sl-UC plants (Fig 4B). The results suggested a stronger RNAi response in Sl-Ma than in Sl-UC plants and agree with the approx 66% reduction of viral RNA in Sl-Ma plants compared to Sl-UC plants (Fig 2). No hybridization was detected with TYRV, indicating high specificity of the sRNA population for the TSWV-CiPz template.

### Hallmark enzymes of the RNAi pathway are modulated differentially in grafted and non-grafted plants

**Fig 5:**
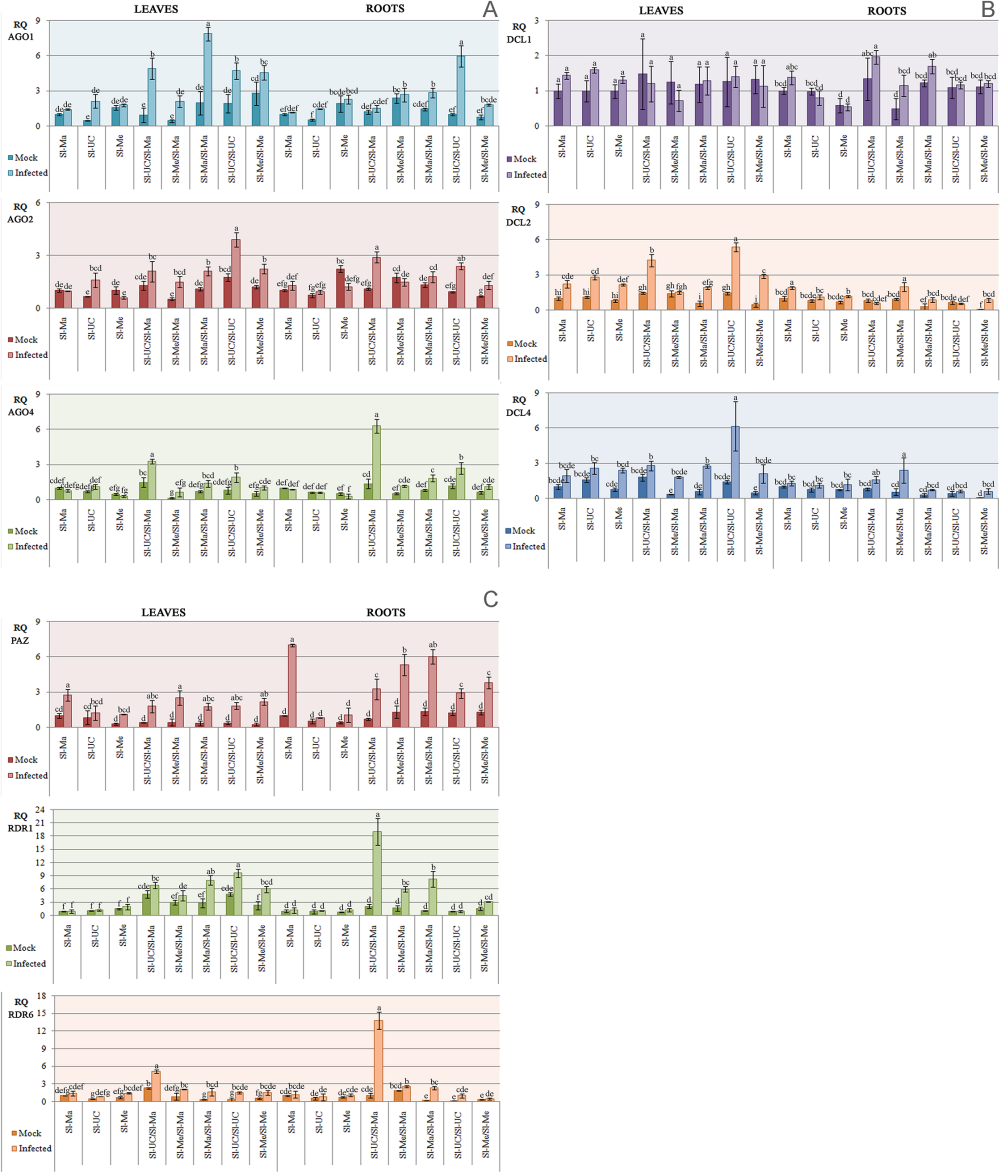
Differential modulation in the expression of RNAi-hallmark enzymes in grafted and non grafted tomato plants. Variations in the expression level of *AGO1*, *AGO2*, *AGO4* (A), *DCL1*, *DCL2*, *DCL4* (B), *PAZ*, *RDR1* and *RDR6* (C) genes (orthologs of *Arabidopsis thaliana*) in leaves and roots of grafted and non-grafted tomato plants at 21 dpi with TSWV-CiPz (infected) or mock-inoculated (mock). For each gene, RQ values are expressed as mean ± standard error of 3 plants (biological replicates) with a technical replicate for each plant. Different letters represent statistically significant differences of means according to factorial analysis of variance (ANOVA) (P ≤ 0.05) (Tukey test). Acronyms for tomato genotypes as in Table 1.

To further substantiate the idea that the observed resistance in Sl-Ma plants compared to Sl-UC, indeed involved a stronger RNAi response, we next analyzed whether any of the hallmark enzymes was transcriptionally upregulated. The relative abundance of *AGO1*, *AGO2*, *AGO4*, *DCL1*, *DCL2*, *DCL4*, *PAZ*, *RDR1* and *RDR6* transcripts (orthologs of *Arabidopsis thaliana*) was estimated by quantitative real-time PCR (qPCR) in leaves and roots of grafted and non-grafted tomato plants, in TSWV-CiPz-challenged and not challenged conditions. Three groups of plants were analyzed: i) non-grafted plants (Sl-Ma, Sl-UC, Sl-Me), ii) plants grafted on Sl-Ma (Sl-UC/Sl-Ma, Sl-Me/Sl-Ma) and iii) self-grafted plants (Sl-Ma/Sl-Ma, Sl-UC/Sl-UC, Sl-Me/Sl-Me). RNA preparations extracted from leaves or roots of mock-inoculated and infected plants collected at 21 dpi were prepared separately from three biological replicates for each condition tested and a technical replicate was included in the test for each biological replicate. Melting curves of each reaction showed a single peak, demonstrating high specificity for primer pairs, while PCR efficiency ranged from 95 to 103%. From the comparison of plants infected by TSWV-CiPz with respective mock-inoculated controls, analysis of the results from qPCR was focalized on genes with fold change ≥ 2 (Fig 5).

In infected non-grafted plants no significant upregulation was observed for any of the genes, with the exception of *PAZ* that was overexpressed in the leaves (2.5-fold) and in the roots (7-fold) of infected Sl*-*Ma genotype, compared to mock-inoculated controls.

In leaves of grafted plants infected by TSWV-CiPZ, a 5.2-fold increase of *AGO1* mRNAs was detected in Sl-UC grafted on Sl*-*Ma rootstock as well as in self-grafted genotypes Sl-Ma/Sl-Ma (4.7-fold) and Sl-UC/Sl-UC (2.4-fold), compared to uninfected controls. A 2.5-fold increase in transcription of *AGO1* was observed also in self-grafted Sl-Me over its non-grafted counterpart, meaning that in this genotype the *AGO1* overexpression was due to the graft itself rather than to a response to viral infection. *AGO2* was overexpressed (2-fold) only in the self-grafted SL-UC/SL-UC genotype and *AGO4* in Sl-UC grafted on Sl-Ma (2.2-fold). A significant overexpression of *DCL2* (3.3-fold) was observed in self-grafted Sl-UC and in Sl-UC grafted on Sl-Ma (2.3-fold) in relation to mock-inoculated control plants and non-grafted plants infected by TSWV-CiPz. Finally, an enrichment of *DCL4* transcripts (4.8-fold) was detected in the infected leaves of self-grafted Sl*-*UC genotype, while *RDR1* was upregulated significantly in those of self-grafted Sl*-*Ma (2.4-fold) and Sl-Me (2.5-fold) genotypes infected by TSWV-CiPz.

In infected root samples from grafted plants, *AGO1* was over-expressed (6-fold) in self-grafted Sl*-*UC genotype and transcripts of *AGO2* and *AGO4* were enriched 3- and 4.5-fold respectively, in Sl*-*UC grafted on Sl*-*Ma. No upregulation of the enzymes was observed in self-grafted plants. *DCL2* showed a 2-fold overexpression in Sl-Ma and Sl-Me grafted on Sl*-*Ma, while PAZ was upregulated (3.3-fold) in Sl-Me/Sl-Ma plants and up to 4.4-fold in Sl-Ma self-grafted plants. Increases in PAZ transcription were also observed in Sl-UC/Sl-Ma (4.7-fold) as well as in Sl-UC (2.3-fold) and Sl-Me (2.3-fold) combinations when compared to not-infected controls. Finally, transcription of *RDR1* was upregulated in Sl-UC/Sl-Ma (8-fold) and Sl-Me/Sl-Ma (3.5-fold) as well as in self-grafted Sl-Ma (7.3-fold), while the upregulation of *RDR6* was observed only in Sl*-*UC/Sl-Ma (12.5-fold) and in self-grafted Sl*-*Ma (12-fold) plants infected by TSWV-CiPz.

### VIGS reveals different ability between Sl-Ma and Sl-UC genotypes in holding an RNA silencing status

**Fig 6:**
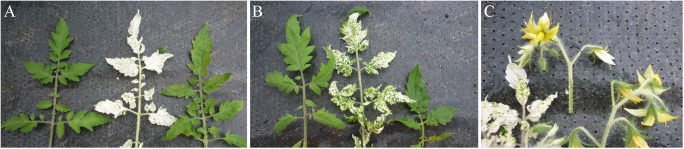
Photo-bleached phenotype is differentially expressed in Sl-Ma and Sl-UC plants. Photo-bleached phenotype induced in Sl*-*Ma (A) and Sl*-*UC (B) at 10 dpa with *A*. *tumefaciens* carrying pTRV1+ pTRV2-PDS. In each panel, helathy (left), agroinfiltrated with *A*. *tumefaciens* carrying pTRV1+ pTRV2-PDS (central) and agroinfiltrated with *A*. *tumefaciens* carrying empty plasmids (right). Panel (C) shows photo-bleached phenotype induced in stem and flower sepals of Sl*-*Ma at 30 dpa.

So far all experiments indicated that Sl-Ma exhibits a stronger RNAi-based response to infection with TSWV-CiPz than Sl-UC. To further validate these findings, an experiment was performed to analyze the rate of silencing during a virus-induced gene silencing (VIGS) test. To this end, a recombinant viral vector based on *Tobacco rattle virus* (TRV) was used to induce silencing of the tomato endogenous *phytoene desaturase* (PDS), a gene commonly used as a visual marker in VIGS and resulting in a characteristic photo-bleached phenotype that can be easily monitored in plants [33, 34]. As expected, silencing of PDS was obtained after agro-infiltration of pTRV1 and pTRV2-PDS (see Materials & Methods) in cotyledons of three plants of each Sl-Ma and Sl-UC genotypes. Cotyledons infiltrated with empty plasmids only served as mock-agroinfiltration controls. Whereas photo-bleaching appeared in the top leaves of two out of three Sl-Ma plants by 5 days post agro-infiltration (dpa) and persisted beyond 30 dpa, in Sl*-*UC plants photo-bleaching appeared not earlier and between 7 and 10 dpa in the top leaves of all the three plants. In comparison to Sl-Ma (Fig 6A) photo-bleaching was clearly less uniform and consisted of white blotches scattered on the green leaf surface (Fig 6B), which reversed/recovered to a uniform green phenotype by 20 dpa again. Moreover, in Sl-Ma plants the photo-bleaching also appeared on stems and flower sepals (Fig 6C).

**Fig 7:**
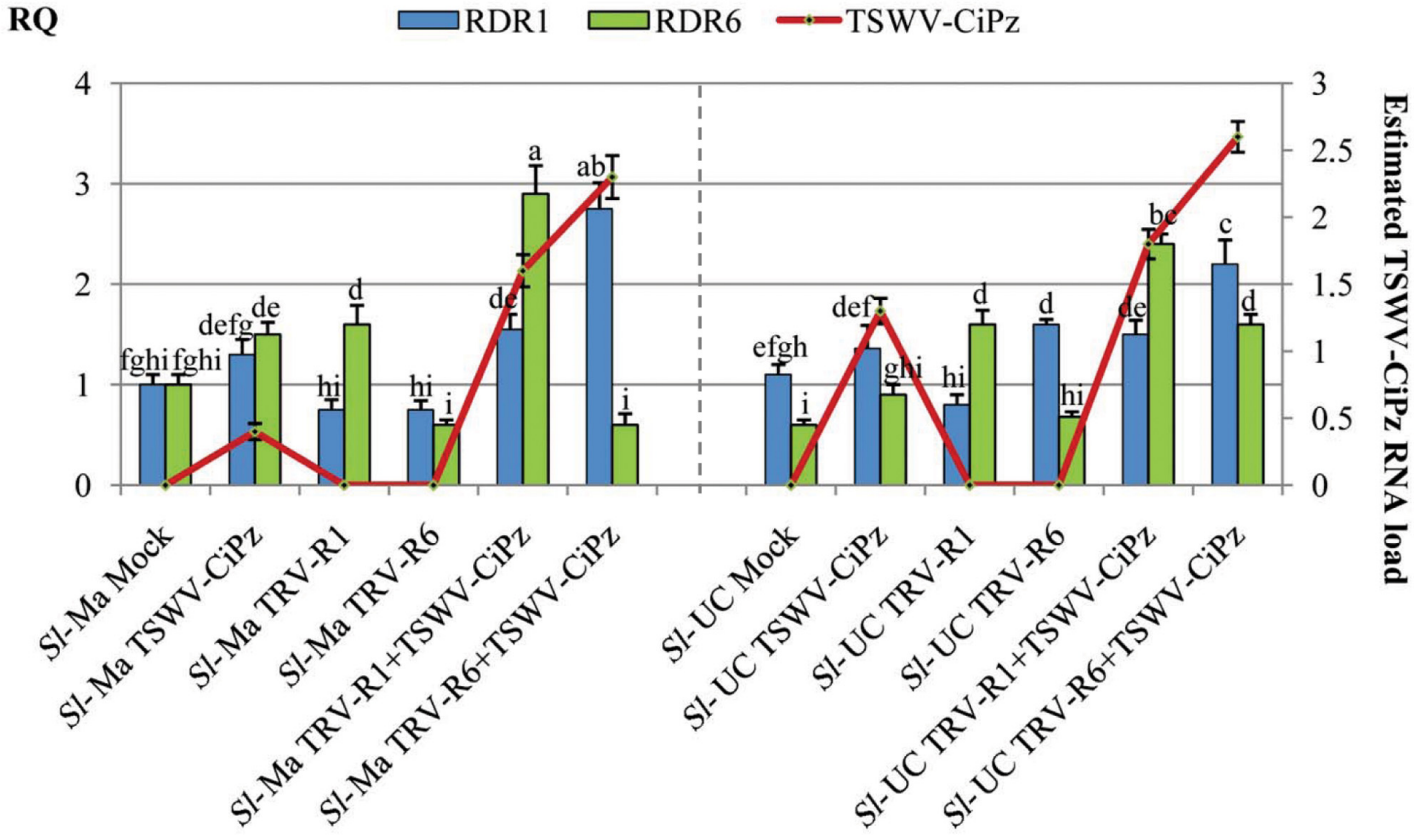
VIGS of RDR1 and RDR6 in Sl-Ma and Sl-UC plants. Relative quantity (RQ) of RDR1 and RDR6 transcripts (columns) in samples of Sl*-*Ma and Sl*-*UC plants collected at 14 dpa with *A*. *tumefaciens* carrying pTRV1+ pTRV2-RDR1 (Sl-Ma TRV-R1 and Sl-UC TRV-R6) and pTRV1+ pTRV2-RDR6 (Sl-Ma TRV-R1 and Sl-Ma TRV-R6). RQ values were first normalized on the accumulation level of the GAPDH mRNA (Δ cycle threshold [Ct] = Ct_GAPDH_–Ct_target_ RNA) and then used to determine the relative quantification of each target RNA with a calibrator, according to the formula ΔΔCt = ΔCt_calibrator_–ΔCt_target_ RNA. Each target mRNA in an individual mock-inoculated plant served as calibrator (RQ set to 1) for the respective gene. RQ for RDR1 and RDR6 transcripts was deduced by the formula expression 2^-ΔΔCt^. Columns represent mean RQ values from three biological replicates and different letters represent statistically significant differences values according to separate one-way ANOVA analysis for each target mRNA, using Tukey’s test (P = 0.05). Vertical bars on columns represent standard deviations among replicates. Figure shows also estimates of the accumulation of TSWV-CiPz RNA (red line) in agroinfiltrated plants. After collection of leaf samples, plants were inoculated with TSWV-CiPZ on the first and second true leaves above remnants of cotyledons and load of viral RNA estimated at 19 dpi with dot blot hybridization. Vertical bars on line represent standard deviations among replicates.

In analogy to the first experiment, a second VIGS experiment was performed but in which instead of PDS, RDR1 and RDR6 were silenced. For this purpose, the PDS gene in pTRV2-PDS was replaced by the cDNAs of *RDR1* or *RDR6* fragments to obtain the pTRV2-RDR1 and pTRV2-RDR6 plasmids, respectively. Plasmids were used for the agro-infiltration of Sl-Ma and Sl-UC genotypes, using three plants for each inoculum. At 14 dpa with pTRV2-RDR1 or pTRV2-RDR6, all plants showed a slightly stunted phenotype in comparison to mock-inoculated controls. The abundance of *RDR1* and *RDR6* transcripts was estimated by quantitative PCR (qPCR) of reverse-transcribed RNA preparations extracted from samples collected at 14 dpa from three distinct plants of Sl-Ma and Sl-UC as well as from their respective controls. Compared to mock-agroinfiltrated plants the results revealed a partial down-regulation in the expression of both genes in the two genotypes that was not significant (Fig 7). However, when these plants were inoculated mechanically with TSWV-CiPz and total RNA extracts prepared at 19 dpi estimated loads of accumulation of viral RNA showed a clear increase in plants agroinfiltrated with pTRV2-RDR1 and pTRV2-RDR6 plasmids. In agreement with previous data, such increase was higher in Sl-UC than in Sl-Ma (Fig 6) indicating that the underlying mechanism for resistance/resistance of Sl-Ma to TSWV-CiPz may involve RDR1 and/or RDR6.

## Discussion

The results of our study show that suitable levels of resistance against an *Sw-5* resistance-breaking strain of TSWV (TSWV-CiPz) can be obtained in tomato by grafting the otherwise susceptible commercial varieties carrying the *Sw-5* gene onto an old tomato variety, denoted Sl*-*Ma. In tests with non-grafted plants, Sl-Ma resisted the TSWV-CiPz infection by limiting viral RNA accumulation and recovering from mild disease symptoms by 21 dpi. The resistance trait was transmitted to susceptible tomato genotypes grafted onto Sl-Ma suggesting that a mobile signal moving through the graft junction could be implicated. The RNAi seemed a good candidate since: i) its signal travels along the vascular system [1], ii) its systemic spread has been revealed for the first time by grafting experiments [35], iii) much of the evidence now available about the movement of siRNAs in plants has been obtained with the same approach [1, 36–38] and iv) it can be transmitted in both directions across a graft junction, although more efficiently from rootstock to scion [36]. From the analysis of sRNAs, hallmark genes involved in RNAi pathway and results from VIGS experiments we found evidence that RNAi plays a role in the resistance showed by Sl-Ma against TSWV-CiPz and in scions grafted on this rootstock. We also found that the graft itself, which was of the type thought to be the most effective in transmitting the silencing signal [39], enhanced the resistance of the scion to viral RNA accumulation and independently from the tomato genotype. This was particularly interesting with self-grafted plants of the Sl-Me variety, which carries the *Sw-5* resistance gene and is susceptible to resistance-breaking strains of TSWV [40]. The resistance mediated by *Sw-*5 is of the gene-for-gene type with *Sw*-5 triggering a hypersensitive reaction around the TSWV infection *foci* [27] and the Nsm fragment of the TSWV genome being the avirulence factor [28]. How self-grafted plants of the otherwise susceptible Sl-Me variety resist infection of an Sw-5 resistance breaking strain of TSWV would require a focused investigation.

In this study we found that all three viral RNAs of TSWV-CiPz were targeted by the population of sRNAs detected in Sl-Ma and Sl-UC but the hybridization signal with the sRNA probe derived from infected Sl*-*Ma plants was higher than that obtained with the sRNA probe from Sl-UC plants. The stronger RNAi response in Sl-Ma than in Sl-UC plants was in good agreement with the 3-fold reduction of viral RNA accumulation in Sl-Ma plants compared to Sl-UC plants. It has been pointed out that a somewhat lower efficiency in the activation of RNAi in the scion could be due to differences in the developmental stage of the plants [39]. However we can rule out this hypothesis since we infected plants at the same developmental stage. We also found that with the exception of *PAZ*, there was no significant upregulation of the other genes in non-grafted infected plants compared to mock-inoculated controls. On the contrary, more enzymes were clearly upregulated in grafted plants challenged with TSWV-CiPz, and among them differences were observed between leaves and roots. Excluding from the analysis enzymes that were upregulated in leaves and/or roots of the self-grafted genotypes, we can conclude that the overexpression of *AGO1*, *AGO4* and *DCL2* in the leaves of Sl-UC grafted on Sl-Ma was very likely in response to viral infection. Similarly, *AGO2*, *AGO4* and *PAZ* were overexpressed in the roots of Sl-UC/Sl-Ma and *DCL2* and *PAZ* in those of Sl-Me/Sl-Ma, while *RDR1* and *RDR6* were both overexpressed in the roots of Sl-UC/Sl-Ma and *RDR1* also in those of Sl-Me/Sl-Ma. Therefore the increased level of transcription of all of these enzymes in Sl-UC and Sl-Me genotypes when grafted on Sl-Ma seemed to be genuinely due to a viral challenge in the grafted setting (Fig 5). The results were in agreement with the observation of symptom attenuation and accumulation of viral RNA in plants grafted on the Sl-Ma genotype and supported the proposal that the resistance of Sl-Ma to TSWV-CiPz could involve an RNAi-based response through the upregulation of the transcription of *AGO2*, *AGO4*, *RDR1* and *RDR6* in the roots of infected plants grafted on Sl-Ma.

Altogether, the results presented here provide a potentially useful approach to mitigate economical losses due to infections of *Sw-5* resistance-breaking strains of TSWV in tomato crops. The genetic uniformity of tomato plants exhibiting the *Sw-5* resistance gene and their wide use by farmers soon after their implementation very likely facilitated the emergence of RB strains of TSWV, which are now prevalent in tomato cropping areas [15, 16]. We have screened a number of *Solanum* spp genotypes and tomato varieties discovering that the Sl*-*Ma variety has traits of resistance to the infection of the *Sw*-5 resistance-breaking strain of TSWV used in this study. Even more interestingly, the grafting exacerbated this characteristic. From an applicative point of view the most encouraging combination is to graft a commercial tomato variety carrying the *Sw-5* gene, like Sl-Me used in this study, onto a tomato variety, like Sl-Ma, with traits of resistance to the infection of *Sw-5* resistance-breaking strains of TSWV. Thus a scion variety with improved RNAi-based characteristics resulting from the transportation of an RNAi signal from the rootstock could be an alternative that might be economically interesting for the growers. As in the case of other grafted vegetables, major costs for the grafting can be compensated by: i) the reduced number of plants transplanted in the field and ii) the major production and better fruit quality of each plant [41].

## Materials and Methods

### Virus source, plant material and grafting procedure

A resistance-breaking isolate of *Tomato spotted wilt virus* (TSWV-CiPz) was collected in Apulia (southern Italy) from chicory plants with necrotic symptoms characteristic of tospoviral infection. The virus was transferred and propagated in *N*. *benthamiana*, by rubbing leaves with sap obtained from leaf tissues of naturally infected chicory plants ground in 100 mM phosphate buffer, pH 7.2, containing 1 mM sodium sulphite. TSWV-CiPz was characterized for the ability to overcome the resistance gene *Sw-5* by mechanical inoculation onto *Solanum lycopersicum* cv Messapico, a commercial tomato variety carrying the *Sw-5* resistance gene but susceptible to TSWV-RB strains [40].

The following wild *Solanum* spp and local ecotypes of tomato and eggplant were provided by an Apulian nursery Plant and tested for susceptibility/resistance to TSWV-CiPz: *Solanum melongena* cv molfettese (Sm-Mo), *S*. *integrifolium*, *S*. *nigrum* (black nightshade), *S*. *torvum* (Turkey Berry), *S*. *lycopersicum* cv manduria (Sl-Ma) and *S*. *lycopersicum* cv regina (Sl-Re). The commercial tomato varieties Messapico (Sl-Me) and Faino (Sl-Fa), carrying the *Sw-5* gene and the susceptible Pullrex (Sl-Pu) and UC82 (Sl-UC) varieties were used in different graft combinations, including self-grafting. Grafting was carried out when the tomato seedlings had two to four true-leaves and the stems were 1.5 to 3 millimeters in diameter. To grow different cultivars to the same developmental stage, sowing time was adjusted according to the germination time required from each plant species/variety. The graft, also denoted top grafting [39], was made by cutting the bottom of the scion into a thin, narrow wedge (“V” shaped), which was inserted into the perpendicular cut done on flat T-shaped cut surface of the rootstock. The scion and the rootstock were clamped together with a silicon clip and plants were incubated under a polyethylene bag to maintain humidity and reduce water loss by transpiration. After two to four days in the healing chamber the leaves of the scions recovered turgor and the plastic bag was removed. Plants were inoculated mechanically on the first leaf above the graft junction within one week after grafting, with sap extracted from leaves of *N*. *benthamiana* plants infected systemically with TSWV-CiPz. All the plants were grown and maintained in a temperature-controlled glasshouse at 24±2°C with 16 h photoperiod and monitored daily for symptom appearance.

### RNA extraction and analysis

Samples for dot blot analysis were collected from inoculated and mock-inoculated plants at 7, 14, 21, 28 days post inoculation (dpi), unless reported otherwise and ground in the presence of 6 vol (w/v) of 50 mM NaOH, 2.5 mM EDTA. The extract was incubated at room temperature for 5 min, then 5 μl were spotted onto a positively charged nylon membrane (Roche Diagnostics, Mannheim, Germany) and nucleic acids cross-linked for 5 min under UV light. Membranes were hybridized overnight at 58°C in 150 μl/cm^2^ of DIG Easy Hyb Granules solution (Roche Diagnostics, Mannheim, Germany) containing 100 ng/ml of DIG-labeled RNA probe of TSWV M RNA synthesized as described previously [42]. After hybridization, probe excess was removed by three washes of 30 min each with 0.1X SSC containing 0.1% SDS (15 mM NaCl, 1.5 mM Na citrate, pH 7, containing 0.1% SDS) followed by washes and hybrid detection according to the instructions of the DIG luminescent detection kit and CDP-star substrate (Roche Diagnostics, Mannheim, Germany). ChemiDoc system apparatus and Quantity One software (Bio-Rad Laboratories) were used to detect and quantify the chemiluminescent hybridization signal (Bio-Rad Laboratories) by using *Glyceraldehyde 3-phosphate dehydrogenase* (GAPDH) as housekeeping gene for normalization [43].

For tissue-print hybridization, sections of roots, stems and leaves of infected and mock-inoculated tomato plants were collected at 19 dpi and cut surfaces gently pressed onto positively charged nylon membrane (Roche Diagnostics, Mannheim, Germany) pre-wetted with 50 mM NaOH, 2.5 mM EDTA and air-dried for 5 min before printing. Viral RNA distribution and accumulation was estimated by hybridization and densitogram analysis as described above.

### Transcript profiling of hallmark enzymes involved in RS

Relative abundance of *AGO1*, *AGO2*, *AGO4*, *DCL1*, *DCL2*, *DCL4*, *PAZ*, *RDR1*, *RDR6* transcripts was estimated by reverse-transcription real-time quantitative PCR (qPCR) using the comparative cycle threshold (2^-ΔΔCt^) method corrected for PCR efficiencies [44]. GAPDH was used as housekeeping gene for normalization of *DCL2*, *DCL4*, *PAZ*, *RDR1* and *RDR6* gene expression, while *β-Tubulin* (TUB) was used for the normalization of *AGO1*, *AGO2 AGO4* and *DCL1*. First-strand cDNA was synthesized from 1 μg of total RNA preparations extracted from samples collected from grafted and non-grafted genotypes at 21 dpi with TSWV-CiPz or buffer (mock-inoculated controls). High Capacity cDNA Reverse Transcription kit (Applied Biosystems, Foster City, CA, USA) and 10 pmol random hexamers were used, following the protocol of the manufacturer. qPCR was set up in 10 μl of 2X Fast SYBR Green PCR Master Mix (Applied Biosystems), containing 100 ng of first strand cDNA template, and 200 nM each of the forward and reverse primer pairs listed in Table 2. Each cDNA sample was amplified in triplicate on a single 48-well optical plate using the StepOne Real-Time PCR system (Applied Biosystems). The cycling profile consisted of 95°C for 20 sec followed by 40 cycles of 3 sec at 95°C and 30 sec at 60°C. After the final PCR cycle, a melting curve analysis was done to determine the specificity of the reaction. Validation experiments were done according to the manufacturer’s instructions (Applied Biosystems) to compare the amplification efficiencies of *AGO1*, *AGO2 AGO4*, *DCL1*, *DCL2*, *DCL4*, *PAZ*, *RDR1*, *RDR6* with that of the respective endogenous mRNA. The experiment was repeated twice and statistical significance of the RQ values was assessed by factorial analysis of variance (ANOVA) (Statistica 7.0, Stat Soft, Inc.1984-2004) with Tukey post-hoc test (P ≤ 0.05), for each target gene, separately.

**Table 2:** List of primer pairs.

Primer names		Primer sequences 5'-3'	Amplicon lengths (bp)	Sources/References
***AGO1***	For	GGAATTGCTGATTTCCTTCCGTCG	164	[48]
	Rev	CTGATAGTTGGGTTCTAAAGATGCAC		
***AGO2***	For	TCTAATGAGCACCTGCCCGA	105	LOC101249141
	Rev	TAAGCACAACGCAAGCCCTC		
***AGO4***	For	TGTGGCTCCGATAAGTTATGCCCA	166	[48]
	Rev	TGGAGCTAGCAACGTTCTCCTGAA		
***DCL1***	For	ACTGTATCGATGTGTGCACGAG	109	[48]
	Rev	GAGTTCCAATAGAAGAGCTGCTG		
***DCL2***	For	GGGACTGTCTCCAGGACTAATA	108	LOC101256737
	Rev	GCATGAAGGATGTGCTTGTG		
***DCL4***	For	GACTTGGTGGAGTCTTGTATGG	102	LOC101055595
	Rev	GCTCATGACGGGCTTTAAGA		
***PAZ***	For	GCACATTCTTCATGCCTCCCAAG	157	SGN-U213736
	Rev	AGTAGTTTCAGCTTCCCATCCG		
***RDR1***	For	ATGCTGAGGCCATTAGTGTTGCTG	107	Y10403
	Rev	CCAAGCCGAAGCCTTTGGTAACAT		
***RDR6***	For	GCGGCTATAATGTTGAGTGCAGGG	106	AP009260
	Rev	GTCTTATTCCTGAGGTCGCCAAGC		
***GAPDH***	For	ACCACAAATTGCCTTGCTCCCTTG	110	[44]
	Rev	ATCAACGGTCTTCTGAGTGGCTGT		
***TUB***	For	CCTGACAGCTTCTGCCATGT	160	[48]
	Rev	CATCTTCAGCCCAGTTGGTG		
**RDR1-vigs**	For	TCCCGCTGAAATACCATCTC	329	AK321474
	Rev	GCCTTCATAATGCCACCACT		
**RDR6-vigs**	For	GTTTTCGAGCACATGGAGC	196	AP009260
	Rev	TAATCTGCTGCAATCCATGC		
**RDR1-*att*B**	For	GGGGACAAGTTTGTACAAAAAAGCAGGCTTATCCCGCTGAAATACCATCTC	390	[34]
	Rev	GGGGACCACTTTGTACAAGAAAGCTGGGTAGCCTTCATAATGCCACCACT		
**RDR6-*att*B**	For	GGGGACAAGTTTGTACAAAAAAGCAGGCTTAGTTTTCGAGCACATGGAGC	257	[34]
	Rev	GGGGACCACTTTGTACAAGAAAGCTGGGTATAATCTGCTGCAATCCATGC		

### VIGS for *PDS*, *RDR1* and *RDR6* and screening of silenced genotypes

Two *A*. *tumefaciens* cultures containing recombinant plasmids pTRV1 and pTRV2-PDS derived from *Tobacco rattle virus* (TRV) were prepared and co-agroinfiltrated in cotyledons of the selected tomato genotypes as described below. For the construction of pTRV2-RDR derivatives, specific primers were designed on conserved regions of *RDR1* (GeneBank Acc. AK321474.1) and *RDR6* (GeneBank Acc. AP009260.1) (Table 2). First-strand cDNAs were reverse-transcribed from 1 μg of a total RNA preparations extracted from 100 mg of leaves of a 1:1 mixture of Sl-Ma and Sl-UC using SuperScript RT (Invitrogen, Carlsbas, CA) in the presence of 20 μM of reverse primers, according to the manufacturer’s instructions. *RDR1* and *RDR6* cDNAs were PCR-amplified in a reaction volume of 25 μl by 3 min denaturation at 95°C followed by 35 cycles of 30 sec denaturation at 95°C, 30 sec annealing at 54°C and 30 sec synthesis at 72°C. Final elongation step was for 7 min at 72°C. Amplicons were eluted from the gel by using homemade spin-columns [45], cloned into pGEM-T Easy Vector (Promega Corp., Madison, WI, U.S.A.) according to the protocol supplied by the manufacturer and used to transform *E*. *coli* DH5α competent cells by applying 2.5 kV with 200 Ω resistance and 25 μF capacitance with a Gene Pulser and Pulse Controller Apparatus (Biorad, UK). Electroporated cells were plated on Luria-Bertani (LB) medium, and selected with standard ampicillin and white/blue colony screening. After sequencing to confirm their identity, RDR1 and RDR6 clones were used to generate recombinant vectors for VIGS as described by Liu and colleagues [34], using the Gateway technology (Invitrogen, Carlsbad, CA) and *att*B primer pairs for BP clonase. The cDNA fragments were subsequently transferred to the destination vector pTRV2 by LR clonase and used to transform *E*. *coli* DH5α competent cells. Vectors pTRV2-RDR1 and pTRV2-RDR6 were electroporated into competent cells of *A*. *tumefaciens* GV3101, positive clones were identified by PCR and used for agroinfiltration.

Cultures of pTRV1 and pTRV2-derivatives (-Empty vector, -PDS, -RDR1 and -RDR6) collected from individual colonies were grown overnight at 28°C in 5 ml of LB3 medium (0.5% yeast extract, 1% peptone, 0.4% NaCl, 0.1% KCl 0.3% MgSO_4_, containing antibiotics 25 μg·ml^-1^ rifampicin, 50 μg·ml^-1^ kanamycin). Cell culture was collected by centrifugation at 3000 g for 20 min, washed once with cold 10 mM MgCl_2_, resuspended in infiltration medium (10 mM M gCl_2_, 10 mM morpholine ethanesulfonic acid pH 5.5, 150 μM acetosyringone) to an optical density at 600 nm of 1.5 and incubated at 28°C for 4 h. pTRV1and pTRV2-derivatives were mixed with a 1:1 ratio and used to infiltrate lower epidermis of tomato cotyledons using a 2.5 ml syringe.

Silencing of endogenous *PDS* in plants agroinfiltrated with pTRV1 and pTRV2-PDS was deduced by the appearance of typical photo-bleached phenotype while the extent of silencing of *RDR1* and *RDR6* was deduced from an estimate of the relative abundance of *RDR1* and *RDR6* transcripts in agroinfiltrated and non agroinfiltrated plants by qPCR, using the primer pairs listed in Table 2 and *GAPDH* as endogenous reference gene. One μg of total RNA preparations were reverse transcribed with Tetro cDNA synthesis kit (Bioline Reagents ltd, UK) in the presence of random primer hexamer mix and then amplified with 2 min denaturation at 95°C followed by 40 cycles of 5 sec denaturation at 95°C, 15 sec annealing and synthesis at 60°C in SensiFAST SYBR-HiROX mix (Bioline Reagents ltd, London, UK) containing 20 μM primers.

### Small RNA purification and mapping on tospovirus genome

Total nucleic acid was extracted from 1 g of leaves collected from Sl-Ma and Sl-UC tomato plants mock-inoculated and at 21 dpi with TSWV-CiPz, according to the method of Bucher et al. [46]. Low-molecular-weight (LMW) RNAs were purified from the mixture of total nucleic acid extracts, by precipitation with 10% PEG 8000 containing 1M NaCl and 200 mM EDTA, pH 8, followed by ethanol precipitation and resuspension in RNase-free water. sRNAs were purified from LMW RNA preparations following the protocol of Haley et al. [47]. During all steps, quality and integrity of each RNA preparation was monitored by spectrophotometric readings with ND-100 Nanodrop (Nanodrop Technologies, Rockland, DE, USA) and gel electrophoresis. For the mapping of sRNAs population on tospoviral genome, sRNAs were labeled with (γ-^32^P) ATP by T4 polynucleotide kinase, according to the manufacturer’s instructions (Perkin Elmer Inc., UK). TSWV-CiPz RNA was extracted from virus particles purified from systemically infected leaves of *N*. *rustica* according to the protocol of Feldhoff et al. [31] while RNA of *Tomato yellow ring virus* (TYRV) was prepared from virus particles purified from leaves of *N*. *benthamiana* following the protocol of Hassani-Mehraban et al., [32]. RNA from both viral species was extracted using TRIzol^®^ reagent (Invitrogen Ltd, Paisley, Renfrewshire, UK) and resuspended in RNase-free water. Equimolar amounts (0.5 μg) of TSWV-CiPz and TYRV RNA were resolved on 1% agarose gel in 0.5X TBE (44.5 mM Tris base, 44.5 mM Boric acid, 1 mM EDTA, pH 8), blotted to Hybond-N membrane (Amersham Biosiences Limited, UK) by a TurboBlotter™ s (Whatman, England) and cross-linked by UV light. One μg of total nucleic acid and 1 μg of sRNA preparations were also run on the gel and used as positive controls. Four replicates of filters were, respectively, hybridized overnight at 48°C with the Sl-Ma and Sl-UC γ-^32^P-labeled sRNAs purified from healthy and infected tomato plants in 360 mM Na_2_HPO_4_, 140 mM NaH_2_PO_4_, containing 7% (w/v) SDS and 1 mM EDTA. After hybridization, membranes were washed three times at 48°C with 2X SSC (0.3 M NaCl, 30 mM Na citrate, pH 7), followed by three washes in 2X SSC, containing 0.2% SDS. To detect hybridization signals, membranes were exposed to a phosphor screen and visualized by phosphoimaging (Molecular Dynamics Typhoon Phosphorimager, Amersham biosciences).
